# Optimized Surface Characteristics and Enhanced in Vivo Osseointegration of Alkali-Treated Titanium with Nanonetwork Structures

**DOI:** 10.3390/ijms20051127

**Published:** 2019-03-05

**Authors:** Yuhao Zeng, Yuanyuan Yang, Luyuan Chen, Derong Yin, Honghao Zhang, Yuichiro Tashiro, Shihoko Inui, Tetsuji Kusumoto, Hiroshi Nishizaki, Tohru Sekino, Joji Okazaki, Satoshi Komasa

**Affiliations:** 1 Department of Removable Prosthodontics and Occlusion, Osaka Dental University, 8-1, Kuzuhahanazono-cho, Hirakata-shi, Osaka 573-1121, Japan; zeng-yuhao@hotmail.com (Y.Z.); yangyuanyuan0801@outlook.com (Y.Y.); y.d.r.nld@hotmail.com (D.Y.); joecheung_asuka@hotmail.com (H.Z.); tashiro@cc.osaka-dent.ac.jp (Y.T.); clair.de.lune.peridota@gmail.com (S.I.); joji@cc.osaka-dent.ac.jp (J.O.); komasa-s@cc.osaka-dent.ac.jp (S.K.); 2 Faculty of Health Sciences, Osaka Dental University, 1-4-4, Makino-honmachi, Hirakata-shi, Osaka 573-1144, Japan; kusumoto@cc.osaka-dent.ac.jp (T.K.); nisizaki@cc.osaka-dent.ac.jp (H.N.); 3 The Institute of Scientific and Industrial Research, Osaka University, Suita, Osaka 565-0871, Japan; sekino@sanken.osaka-u.ac.jp

**Keywords:** implant, alkali treatment, in vivo study, nanonetwork, osseointegration

## Abstract

Alkali-treated titanium (Ti) with a porous, homogeneous, and uniform nanonetwork structure (TNS) that enables establishment of a more rapid and firmer osteointegration than titanium has recently been reported. However, the mechanisms underlying the enhanced osteogenic activity on TNS remains to be elucidated. This study aimed to evaluate the surface physicochemical properties of Ti and TNS, and investigate osteoinduction and osteointegration in vivo. Surface characteristics were evaluated using scanning electron microscopy (SEM), scanning probe microscopy (SPM), and X-ray photoelectron spectrometry (XPS), and the surface electrostatic force of TNS was determined using solid zeta potential. This study also evaluated the adsorption of bovine serum albumin (BSA) and human plasma fibronectin (HFN) on Ti and TNS surfaces using quartz crystal microbalance (QCM) sensors, and apatite formation on Ti and TNS surfaces was examined using a simulated body fluid (SBF) test. Compared with Ti, the newly developed TNS enhanced BSA and HFN absorbance capacity and promoted apatite formation. Furthermore, TNS held less negative charge than Ti. Notably, sequential fluorescence labeling and microcomputed tomography assessment indicated that TNS screws implanted into rat femurs exhibited remarkably enhanced osteointegration compared with Ti screws. These results indicate that alkali-treated titanium implant with a nanonetwork structure has considerable potential for future clinical applications in dentistry and orthopedics.

## 1. Introduction

Due to increasing populations and the aging of our society, the demand for dental and orthopedic implants is growing at a rapid rate [1]. In the United States (US), approximately one-third of the populations older than 65 years are fully edentulous, and require dental implants to replace missing teeth [2]. Furthermore, in the US, more than 500,000 procedures are performed each year for hip and knee replacements [3]. Although titanium and its alloys, which have superior biocompatibility, high mechanical resistance, and excellent corrosion resistance, have been commonly used in the manufacturing dental devices and orthopedic surgery [4], the application of titanium implants is limited due to the risk of postoperative infections, protracted healing time (4–6 months) of the implant-bone integration, and insufficient osteoconductivity, particularly in patients with osteoporosis [5]. In this regard, Simonis et al. have reported that the titanium implant survival rate is 89.23% after a 10-year observation period and 82.94% after a 16-year observation period [6].

To enhance the therapeutic indications of titanium implants and improve the biological capability of titanium, it is imperative to explore effective approaches that ensure the more rapid and firmer osteointegration of titanium implants [1,7,8].

It is presently known that implant surface physical and chemical characteristics are essential factors affecting the rate and extent of osteointegration [9,10]. In terms of surface physical characteristics, recent studies have highlighted that nanostructure titanium may have better osteointegration than titanium with microscale surface modifications because nanoscale surfaces have a larger surface area and may better mimic the extracellular matrix to facilitate rapid bone accrual and, hence, promote the adsorption of proteins, cell adhesion and proliferation, gene regulation, and tissue integration [11,12]. Moreover, some studies have reported that nanostructures on titanium surfaces can also alter RGDS (arginine-glycine-aspartic acid-serine) conformations to promote cell adhesion, and these RGDS are likely to be more exposed on nanoscale surfaces than on other types of surface [13]. A further study has suggested a higher adsorption of fibronectin on nanostructure surfaces, which ultimately promotes human osteoblast-like cell attachment [14]. Previously, efforts have been made to obtain these advanced nanoscale surfaces and various novel techniques have been applied in the manufacture of different nanoscale titanium surfaces, including physical methods such as self-assembly of monolayers [14] and ion beam deposition; chemical methods, such as peroxidation (H_2_O_2_) [15,16], acid etching [17], sol-gel transformation of nano-particle deposition [18], and pulsed laser deposition [19], pulsed plasma deposition (PPD) [20], and optical methods [21,22].

Over the past 10 years, alkali treatment of titanium and its alloys, which can produce complex surface nanoscale structures, has attracted considerable attention. Kim et al. [23], for example, were the first to demonstrate alkali and heat treatment of the surface of titanium and indicated its favorable bioactivity with observation of bone-like apatite formation on the surface using simulated body fluid (SBF) tests. Furthermore, Nishio et al. [24] reported the higher osteoblastic differentiation of bone marrow cells on the alkali- and heat-treated titanium. In our previous studies [25], a novel method of alkali treatment of titanium was developed to produce porous and homogeneous uniform nanonetwork structures (TNS) characterized by a basic sodium titanate layer on the surface. These TNS have been successfully fabricated in concentrated alkali solution at room temperature. In contrast with numerous other alkali and heat treatments and production methods, the manufacturing process of TNS is considered to be simple and inexpensive, consumes minimal energy, and is environmentally friendly. The reaction conditions are completely controllable and, hence, we are able to produce titanium materials with such surfaces in stable and large amounts. Xing et al. [26] were the first to determine the optimum concentrations of NaOH solutions for producing TNS and suggested its superior advantages with regards to osteogenic activity of rat bone marrow mesenchymal stem cells (rBMMSCs). Furthermore, TNS has been successfully introduced on titanium implant screws, and has been shown to enhance osteointegration in vivo tests [27]. Therefore, the improved alkali treatment of titanium has considerable potential as an advanced technique for the modification of titanium surfaces and may appreciably enhance the clinical performance of titanium in bone implants.

To date, however, the mechanism underlying the enhanced osteogenic activity on TNS remains to be elucidated. In this study, these underlying mechanisms have been investigated using three different forms of TNS materials, namely, TNS discs, TNS screws, and TNS QCM sensors. Discs and screw are commonly used in implant material research, and in this study, we used QCM sensors to evaluate the effects of TNS surfaces on the adsorption of bovine serum albumin and human plasma fibronectin. Furthermore, we analyzed the surface electrostatic force of TNS using solid zeta potential and examined the ability of apatite formation in SBF tests. In order to investigate in vivo biocompatibility, we examined the performance of Ti and TNS implants in male Sprague–Dawley rats. We anticipate that the results of this research will provide convincing evidence for the future clinical application of TNS and the development of a wider range of applications. 

## 2. Results

### 2.1. Sample Preparation

**Figure 1:**
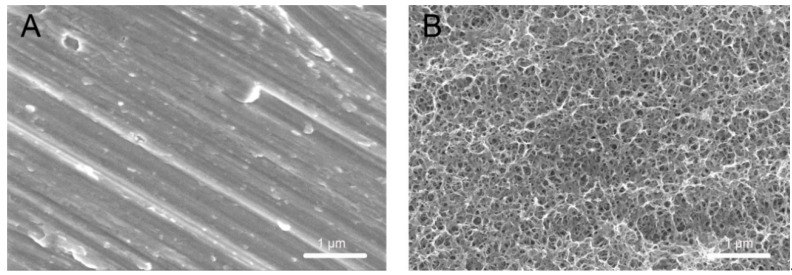
Scanning electron micrographs of (**A**) Ti and (**B**) TNS.

Scanning electron microscopy (SEM) micrographs revealed that the reticular porous network structures at a nanometer level were well interconnected and uniformly distributed on the titanium surface after modifying in 10 M NaOH at 30 °C (Figure 1).

**Figure 2:**
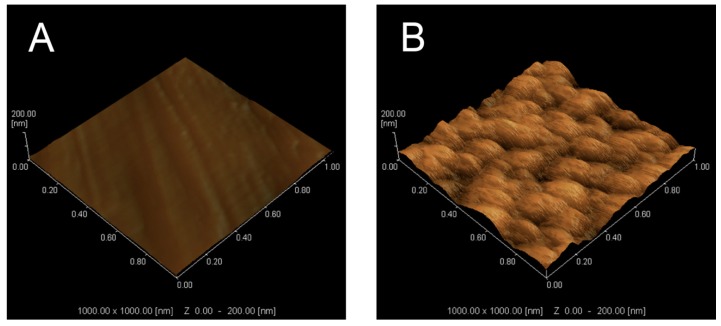
Scanning probe micrographs of (**A**) Ti and (**B**) TNS.

Scanning probe microscopy (SPM) was used to further assess the surface morphology and roughness value (Ra) of the TNS and Ti discs. As shown in Figure 2, the SPM result confirmed the development of a nanoporous network on the TNS and that the Ra was 18.27 nm and 7.07 nm for the TNS and Ti, respectively (Table 1).

### 2.2. Surface Chemical Analyses

**Figure 3:**
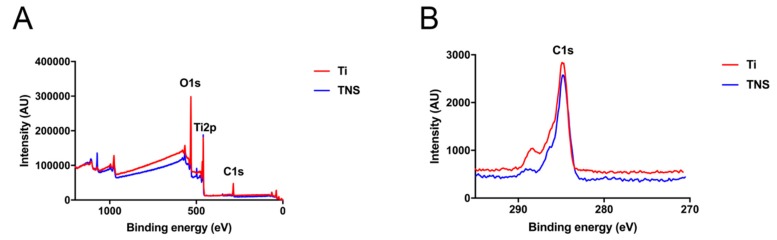
XPS survey spectra of Ti and TNS by wide analysis (**A**) and narrow analysis of C1s (**B**).

The results of the XPS surface chemical analyses of TNS and Ti discs are shown in Figure 3. The Ti2p peak has been observed on the surfaces of both TNS and Ti, indicating that both were covered by titanate. Furthermore, we assumed that the appearance of the Na1s peak on TNS could be attributed to the alkali treatment. Additionally, the C1s peak on TNS was significantly lower than that on Ti, and that the atomic percentage of carbon was decreased on the modified surface (Table 2).

### 2.3. Zeta Potentials 

**Figure 4:**
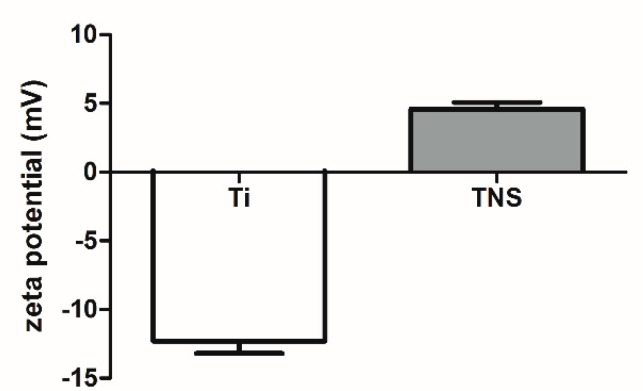
Zeta potential of Ti and TNS (*p* < 0.01).

Figure 4 shows that there were significant differences in the zeta potentials between Ti and TNS. The zeta potential of Ti was negative, whereas that of TNS was positive (*p* < 0.01).

### 2.4. Protein Adsorption Studies

**Figure 5:**
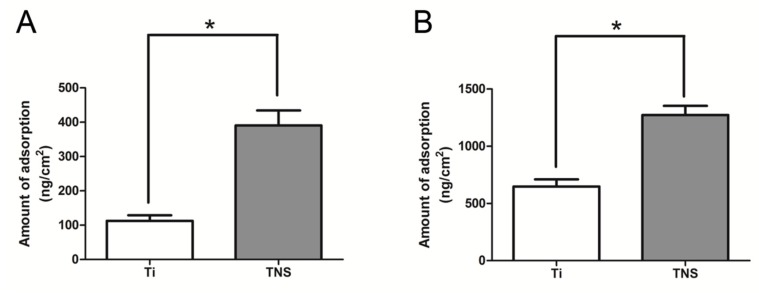
Adsorption of albumin and fibronectin on Ti QCM sensors (**A**) and TNS QCM sensors (**B**).

The adsorption of both albumin and fibronectin on Ti and TNS sensors was determined by QCM measurement (Figure 5). The quantities of albumin adsorbed on the Ti and TNS sensors were 112.5 and 390.75 ng/cm^2^, respectively, whereas the amounts of fibronectin adsorbed on the Ti and TNS sensors were 647.5 and 1272.5 ng/cm^2^, respectively. The amounts of both albumin and fibronectin adsorbed on the TNS sensors were significantly higher than those adsorbed on the Ti sensors.

### 2.5. Simulated Body Fluid Immersion Experiment

**Figure 6:**
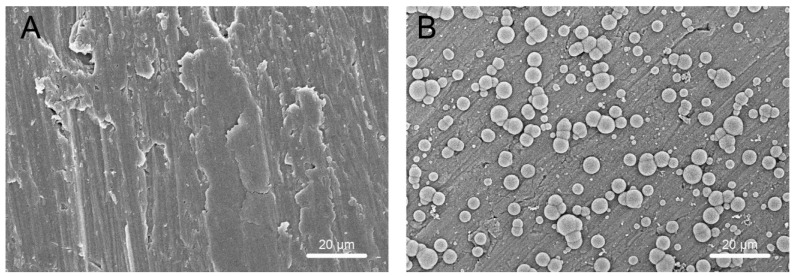
Deposition of apatite on Ti (**A**) and TNS (**B**).

As shown in Figure 6, scanning electron microscopy (SEM) revealed the deposition of apatite on the surface of TNS discs immersed in SBF and maintained at 37 °C for one week. In contrast, little apatite formed on the surface of Ti under the same conditions. The results of SBF thus indicated that TNS has greater bioactivity than Ti.

### 2.6. Micro-CT Assessment

**Figure 7:**
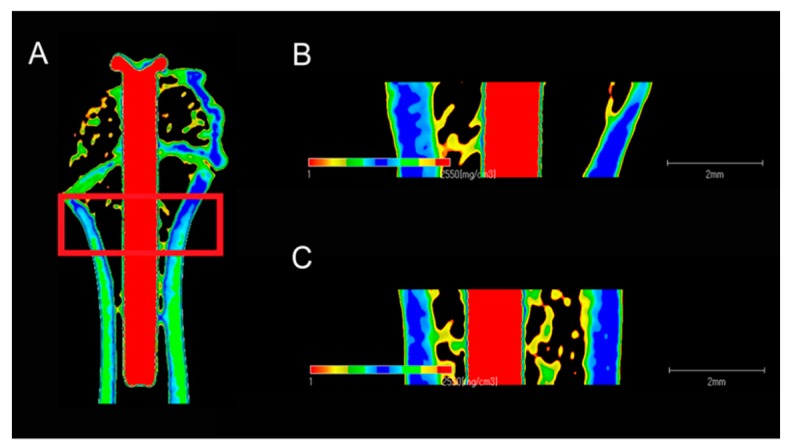
Longitudinal reconstructed microcomputed tomographs (**A**), of Ti (**B**) and TNS (**C**) implants within the region of interest eight weeks after surgery.

**Figure 8:**
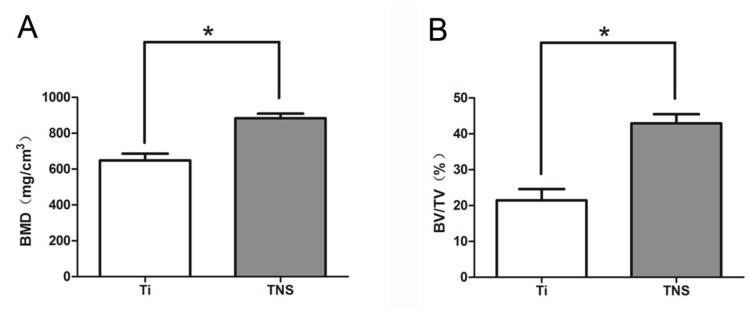
Bone mineral density (BMD) (**A**) of Ti and TNS, Bone volume to total volume ratio (BV/TV) (**b**). * *p* < 0.05.

Figure 7 shows the reconstructed three-dimensional micro-computed tomography image of rat femurs containing implants, in which the cortical bone is indicated by yellow color, the cancellous bone by green color, and the implants by red color. Figure 8 shows the quantitative evaluation of the effects of osteogenesis around the femoral implant within the region of interest (ROI), with the bone volume to total volume (BV/TV) and bone mineral density (BMD) values for the TNS implants being significantly higher than those of the Ti implants (*p* < 0.05). 

### 2.7. Histological and Histomorphometric Evaluations

**Figure 9:**
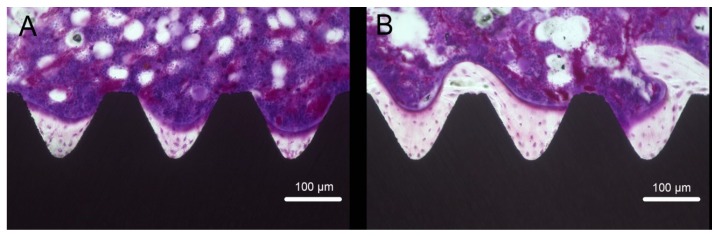
Villanueva staining of bone tissues around Ti (**A**) and TNS (**B**) implants.

**Figure 10:**
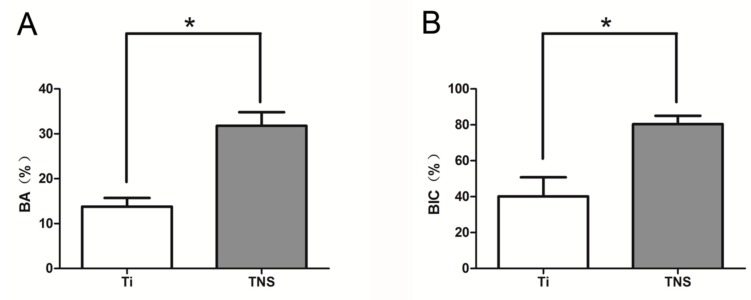
Bone area ratio (BA) (**A**) and bone–implant contact (BIC) (**B**) of Ti and TNS implants. * *p* < 0.05.

Longitudinal sections of regions including the implant and peri-implant bones were used to evaluate the formation of new bone around an implant. The results obtained at eight weeks after surgery showed larger amounts of newly formed bone around the TNS implant surface than that around the Ti implant at the same position (Figure 9). Quantitative histomorphometric analysis of the regions of interest showed that the BA and BIC were significantly greater around the experimental group implants than the control group implants (Figure 10) (*p* < 0.05).

### 2.8. Fluorescence Labeling Analysis

**Figure 11:**
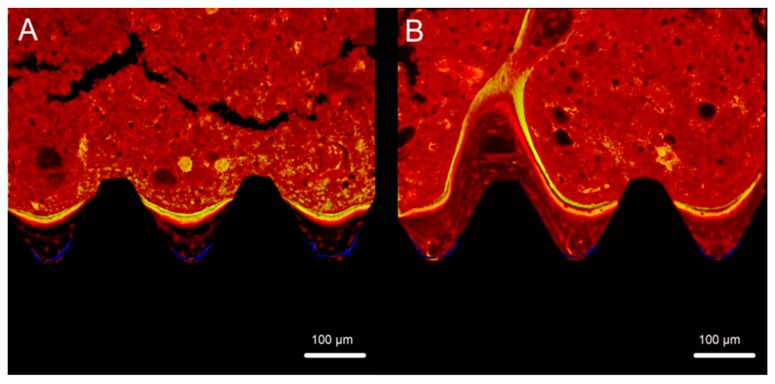
Fluorescence labeling of new bone and mineralization around Ti (**A**) and TNS (**B**) implants.

**Figure 12:**
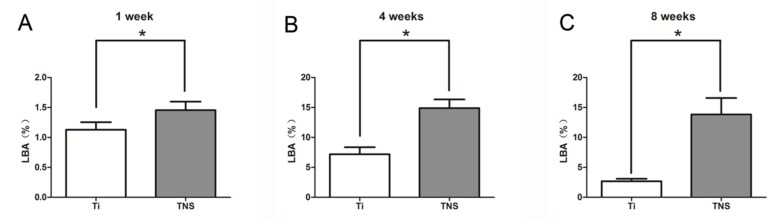
Fluorescently labeled bone area (LBA) after one week (**A**), four weeks (**B**), and eight weeks (**C**). * *p* < 0.05.

Fluorescence labeling facilitated the identification of newly formed bone around an implant at different periods. In this study, oxytetracycline hydrochloride (blue) was used to examine bone formed at one week, alizarin red S (red) for bone formed at four weeks, and calcein (green) for bone formed at eight weeks. Confocal laser scanning microscopy revealed that the labeled bone assessed by between the implant surface and the boundaries labeled at different time points was significantly higher around the experimental group implants than around the control group implants at different observation times (Figure 11 and Figure 12).

## 3. Discussion

Osseointegration is defined by the American Academy of Implant Dentistry as “Contact established without interposition of nonbone tissue between normal remodeled bone and an implant entailing a sustained transfer and distribution of load from the implant to and within the bone tissue.” In clinical terms, osseointegration is also defined as the stability and ankylosis of an implant in bone [28]. The characteristic of the implant surface, including both surface topography and chemical composition, is implicated in the complex process of osseointegration in many different ways [29]. In previous research [25], the cell behavior on titanium surfaces modified by chemical processing at room temperature was examined and the capacity of these modified surfaces to affect the osteogenic differentiation of rBMMSCs was assessed. In the present study, the surface topography and chemistry of TNS were investigated, and then the biological properties of the surface of modified endosseous implants were evaluated based on both in vitro and in vivo analyses.

SEM makes a considerable contribution to the observation and characterization of nanotopography and associated nanostructures on the surface of implants [30]. From the result of high-magnification SEM images, the homogeneous nanoporous network structures on the surface of modified titanium has been observed in the present study, which is consistent with the observations made by Pattanayak et al. [31]. On the basis of the findings from previous studies, it has been established that some of the Ti-O-Ti bonds in the nanoparticles are broken by treatment with NaOH solution to generate Ti-O-Na and Ti-OH bonds [32,33]. This modification of chemical bonds leads to the formation of a Ti-O-Na layer as a consequence of the electrostatic repulsion of the charge on sodium [34]. Compared with the treatment in which nanoparticles or other materials were coated on the surface of implants, there was no bonding interface between the nanostructure and implant owing to the fact that nanostructures were generated by chemical reaction on the surfaces of the materials themselves. Consequently, the risk of the nanostructure becoming detached from substrate materials can effectively be avoided during application. Furthermore, the roughness and topography of modified titanium surfaces also have a significant influence on the wettability behavior [35], and the Ra of the implant surface routinely serves as a height parameter to characterize surface roughness [36]. As revealed by the 3D images obtained using atomic force microscopy (AFM) and the values of Ra, the roughness of the TNS surface was significantly greater than that of the Ti surface, indicating that the TNS surface may promote better cell attachment and growth due to its rougher topography [37], probably by altering the initial macromolecular biological responses. This assumption is consistent with the findings of previous studies [25], which revealed bioactivity on the TNS surface. Furthermore, compared with the Ti surface, chemical analysis indicated a reduction in hydrocarbons on the surface of TNS. It could be accordingly considered that chemical surface modification of titanium endowed its surface with hydrophilic properties and effectively eliminated surface hydrocarbon contamination [38,39], which is consistent with previous observations [40].

Balasundaram et al. [41] have suggested that initial variations in the interaction of proteins and implant surfaces could significantly affect osteoblast adhesion, which is essential for subsequent osseointegration. The results obtained in the present study indicated that the amounts of both fibronectin and albumin adsorbed on TNS sensors were significantly higher than those absorbed on Ti sensors, which may be attributed to the nanoscale topography of the modified surface [29], as evidenced by our SEM and SPM results. Fibronectin, which has RGDS, is a large glycoprotein of the extracellular matrix and plays a crucial role in mediating the adhesion of osteoblasts to implant surfaces [42]. Additionally, albumin, which can be considered as a standard protein, and inhibits the adsorption of proteins that may stimulate inflammation and bacterial colonization adsorbed on implant surfaces [43], is affected by changes in the zeta potential. The zeta potential is the electric potential in the interfacial double layer (DL) at the location of the slipping plane relative to a point in the bulk fluid away from the interface. In this study, the zeta potential is different among the Ti group and the TNS group due to the changes of the surface characteristic after alkali treatment. As a result of electrostatic interaction, negatively-charged proteins that are associated with the enhancement of osseointegration are largely adsorbed to the positively-charged TNS due to their opposite charges [44]. Therefore, nanostructuring increases the wettability of surfaces (determined by contact angle in our previous studies [25]) to blood and the proliferation and binding of fibrin and matrix proteins. Consequently, this could be beneficial for cell attachment and tissue healing, particularly for immediate implantation, which is an important facet of osseointegration.

Furthermore, SEM was used to assess the apatite-forming ability of TNS in SBF. Compared with the titanium surface, formation of large quantities of apatite was prominently observed on the surface of the TNS. In a previous study by Kokubo et al., a material capable of forming apatite on its surface in SBF was found to bond tightly to a living body, as a result of the formation of an apatite layer on its surface in the living body [45]. These findings indicate that the modified implant surface can effectively enhance the promotion of osseointegration, which is consistent with the result of the present animal experiments.

In the present study, micro-CT imaging has been used for the observation of longitudinal sections, and through 3-D modeling analysis we found that bone formation BV/TV and BMD on the TNS surface were better than those on Ti, implying that the formation of new bone induced by the nanostructure surface of TNS is superior to that on the surface of titanium in terms of both quality and quantity. Similarly, compared with titanium implants, the BIC and BA of newly formed bone on the TNS implants was found to be significantly enhanced, thereby indicating that the nanostructure surface of TNS implants efficiently enhances osseointegration capacity within eight weeks due to its more conducive surface properties. The results of the in vivo experiment agree with the findings of previous studies [25], and have demonstrated the influence of TNS in terms of its in vitro bioactivity.

As observed in histological sections, osteogenesis had occurred via osteoinduction on the implant surface, instead of on the distal bone surrounding the implant. Furthermore, time-course analysis by application of fluorescent stains revealed that the amount of new bone formed around the TNS implant had increased significantly after four weeks compared with the titanium implant, thereby further indicating that the TNS surface has an enhanced capacity for the promotion of osseointegration. The enhanced biological performance of the TNS in vivo might be attributable to its superior surface physicochemical properties, as well as the effect of altered expression levels of key osteogenic regulatory genes such as Runx2, which might play important roles in osseointegration via the MAPK pathway [46,47]. 

In this study, three forms of titanium, namely, discs, QCM sensors, and implants, were utilized to conduct a comprehensive evaluation of various aspects of implant performance, which can provide a new concept for future research related to implant materials and surface modification. Further detailed studies are, however, necessary to gain a better understanding of the potential beneficial effects of alkali-treated nanostructure surfaces on osseointegration and biomechanical properties with respect to cortical and corticocancellous bone in larger animals.

## 4. Materials and Methods 

### 4.1. Specimen Preparation 

**Figure 13:**
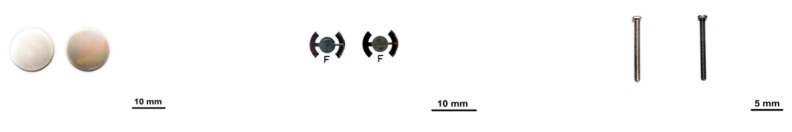
Gross appearance of titanium (Ti) and titanium with nanonetwork structures (TNS) (**A**) discs, (**B**) QCM sensors, and (**C**) screws.

Pure grade 2 commercially available titanium discs (15 mm in diameter and 1 mm in thickness) and screw implants (1.2 mm in external diameter and 12 mm in length) were prepared by machining (Daido Steel, Osaka, Japan) for the evaluation of surface characteristics and animal studies, respectively. The discs were then polished using increasing grades of SiC abrasive paper (600, 800, 1000, and 1500 grits). All the samples were ultrasonically cleaned sequentially with acetone, ethanol, and deionized water (each for 10 min), and then air-dried at room temperature overnight. Ti QCM sensors were fabricated by reactive magnetron sputtering, with a thin layer of Ti deposited on the surface using a radio-frequency magnetron sputtering system (CFS-4ES-231; Shibaura Mechatronics Co., Ltd., Kanagawa, Japan). In order to obtain nanonetwork structures (TNS), half of the titanium samples were immersed in aqueous 10 M NaOH solution at 30 °C for 24 h, and each sample was then rinsed several times with ion-exchanged water until the solution reached a conductivity of 5 μS/cm^3^. Untreated titanium samples (Ti) were used as controls (Figure 13). 

### 4.2. Surface Analysis 

The surface topography of Ti and TNS were observed using scanning electron microscopy (SEM: S-4800; Hitachi, Tokyo, Japan). The mean average surface roughness (Ra) and three-dimensional surface topography of the samples were then assayed under a scanning probe microscope (Shimadzu, Tokyo, Japan). X-ray photoelectron spectrometry (XPS; PHI X-tool; ULVAC-PHI, Kanagawa, Japan) was also performed to evaluate the surface elemental compositions of Ti and TNS.

### 4.3. Zeta Potential

The zeta potentials of both Ti and TNS discs were measured using an ELSZ-1000ZS device (Otsuka Electronics, Hirakata, Japan) at 25 °C. The principle of the measurement is electrophoretic light scattering (laser Doppler electrophoresis), a reliable measurement based on electroosmosis profile estimation.

### 4.4. QCM Measurements

Bovine serum albumin (BSA; Wako Pure Chemical Industries Ltd., Osaka, Japan) and human plasma fibronectin (HFN; Nacalai tesque, Inc., Kyoto, Japan) were used as model proteins to evaluate the effects of protein solutions on TNS surfaces. The amounts of both proteins were determined by QCM measurements (Affinix QN μ; Initium Co., Ltd., Tokyo, Japan). The QCM measurements conducted in this study have been described previously [48]. Briefly, the Ti QCM and TNS QCM sensors were immersed in 500 μL of phosphate-buffered saline (PBS; 0.01 M PBS at pH 7.4), and then the recording started immediately after the infusion of 5 μL (20 μg/mL) BAS or HFN, under constant stirring of the solution to avoid any influence of protein dispersion. Changes in the QCM frequency were measured as a function of time. In accordance with the Sauerbrey equation, the change in frequency was determined by the adsorbed mass.

### 4.5. Simulated Body Fluid Test

For evaluation of apatite formation on the surface of Ti and TNS, the specimens were soaked in 50 mL of SBF solution at 35–38 °C for seven days. The ion concentrations of the SBF solution are comparable to those of human blood plasma [49]. After soaking in SBF solution, all the specimens were rinsed several times in distilled water. Morphological changes in the surface of specimens were analyzed by SEM (S-4800; Hitachi, Tokyo, Japan). 

### 4.6. Animal Model and Surgical Procedures 

**Figure 14:**
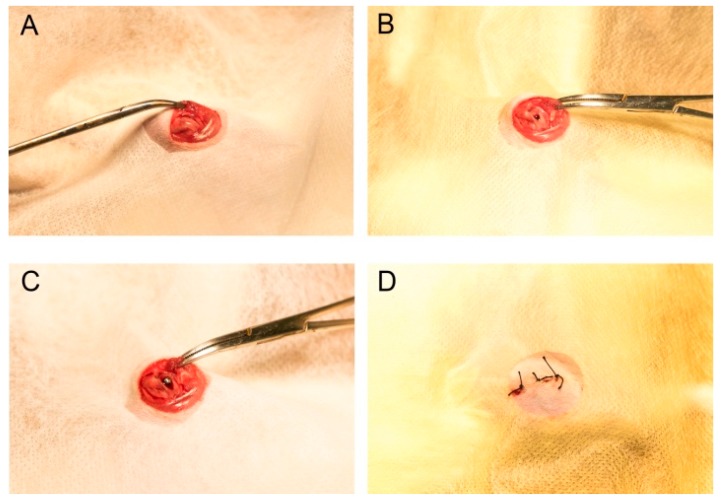
Implantation into rat femurs. (**A**) Incision; (**B**) drilling a hole; (**C**) placement of the implant; and (**D**) closure.

Surgical implantation was performed on twenty-eight-week-old male Sprague–Dawley rats, weighing 180–200 g, for an observation period of eight weeks post-surgery. The in vivo study was conducted in accordance with the ethical principles of the National Animal Care Guidelines and was approved by the Medical Ethics Committee of Osaka Dental University, Japan (approval no. 16-08002, August 2, 2016). After shaving and scrubbing the legs of rats with a mixture of iodine and 75% alcohol solution, the distal aspects of the femurs were carefully exposed via a 10-mm vertical skin incision at the knee joint of the right hind limb and muscle dissection (Figure 14A). Thereafter, under profuse saline irrigation, a 1.2-mm pilot hole was drilled using a 1-mm-round dental bur in the intercondylar notch as the implant placement site (Figure 14B). Subsequently, Ti and TNS screws were inserted into the holes (Figure 14C). Muscle and skin were sutured separately, and the surgical sites were then closed in layers (Figure 14D). To prevent post-surgical infection and reduce postsurgical pain, all rats received intramuscular injections of gentamicin (1 mg/kg) and buprenorphine (0.05 mg/kg) for three days. Following surgery, all the animals were maintained in their normal cages without any restriction.

### 4.7. Sequential Fluorescence Labeling and Micro-Computed Tomography Assessment 

For polychrome sequential labeling of new bone formation and mineralization after implantation, the rats were injected with 30 mg/kg oxytetracycline hydrochloride (Sigma, St. Louis, MO, USA) at 1 week, with 30 mg/kg alizarin red S (Sigma) at four weeks, and with 20 mg/kg calcein (Sigma) at eight weeks. Rats were sacrificed at eight weeks using an intraperitoneal injection of overdose sodium pentobarbital.

The right femurs containing the implants were dissected at eight weeks after surgery, and then immediately maintained in cool saline solution. In an attempt to investigate the effects of implant integration on bone defect repair, the harvested bone specimens were examined using a micro-computed tomography (CT) system (Shimadzu, Tokyo, Japan), running at a voltage 70 kV and current of 118 μA. After scanning, three-dimensional (3D) reconstruction models were generated and analyzed, using morphometric software in Tri/3D-BON, from the region of interest (ROI), defined as that being 2 mm below the highest point of the growth plate and extending 500 μm around implants. The bone volume fraction (BV/TV) and bone mineral density (BMD) were quantified within the ROI. 

### 4.8. Histological and Histomorphometric Analyses 

After performing micro-computed tomography, the femoral specimens were collected and stained using the Villanueva method to assess osseointegration [50]. All sectioning procedures were executed under running water to maintain a cool temperature. A BZ-9000 digital microscope (Keyence Co., Osaka, Japan) and laser scanning microscope (Carl Zeiss, Oberkochen, Germany) were used to analyze the ingrowth of the bone. The excitation/emission wavelengths of the chelating fluorochromes were 351/460 nm for oxytetracycline hydrochloride (blue), 543/617 nm alizarin red S (red), and 488/517 nm for calcein (green), respectively. The bone area ratio (BA), bone-implant contact (BIC), and labeled bone area (LBA) were assessed using ImageJ in a 200× field around the implant and in a section 2 mm below the growth plate for each.

### 4.9. Statistical analyses 

All data are expressed as the mean ± standard deviation. Statistical analyses were performed using Microsoft Excel. Each experiment was repeated three times and similar experiments were carried out in triplicate. *p*-values < 0.05 were considered statistically significant. 

## 5. Conclusions

In this study, a novel implant material was produced using a room temperature alkali treatment technique. This resulted in the generation of samples with rough-surfaced TNS. After soaking in simulated body fluid, TNS implants were observed to form a bone-like hydroxyl apatite. The results of QCM and zeta potential analyses indicated that this TNS surface could lead to faster osseointegration. Our in vivo study results were consistent with the findings of previous in vitro experiments. Analyses of TNS material implanted into an animal model based on micro-CT scanning, histological observations, and polychrome sequential fluorescence labeling, indicated that the TNS implants facilitate continuous induction of osteogenesis and show faster osseointegration than traditional Ti implants. Therefore, we conclude that TNS implants have considerable potential as a bioactive implant material for clinical dental application.

## Figures and Tables

**Table 1:** Roughness values of Ti and TNS.

Device	Ra (nm)
Ti	7.07 ± 0.56
TNS	18.27 ± 2.34

^*^p < 0.05; Ra: roughness values.

**Table 2:** Atomic percentages of elements on Ti and TNS.

	Atomic Percentage (at%)		
Device	Ti2p	C1s	Nals
Ti	19.79 ± 1.41	29.11 ± 0.65	0.04 ± 0.35
TNS	19.69 ± 0.76	25.11 ± 1.24	5.41 ± 2.01

## Abbreviations

Ti	Titanium
TNS	Titanium with nanonetwork structure
SEM	Scanning electron microscopy
XPS	X-ray photoelectron spectrometry
SPM	Scanning probe microscopy
AFM	Atomic Force Microscope
BSA	Bovine serum albumin
HFN	Human plasma fibronectin
rBMMSCs	Rat bone marrow mesenchymal stem cells
RGDS	Arginine-Glycine-Aspartic Acid-Serine
QCM	Quartz crystal microbalance
SBF	Simulated body fluid
RUNX2	Runt-related transcription factor 2
ROI	Region of interest
BMD	Bone mineral density
BV/TV	Bone volume to total volume
BA	Bone area ratio
BIC	Bone–implant contact
LBA	Labeled bone area
